# Persistent mullerian duct syndrome

**DOI:** 10.4103/0971-3026.59761

**Published:** 2010-02

**Authors:** Divya Renu, B Ganesh Rao, K Ranganath, Namitha

**Affiliations:** Department of Radiodiagnosis, RAGAVS Diagnostic and Research Centre Pvt Ltd, Sadguru Complex, No.14, 27^th^ Cross, 4^th^ Block West, Jayanagar, Bangalore - 560 011, India

**Keywords:** Cryptorchidism, inguinal hernia, mullerian inhibiting factor, MRI, mullerian duct derivatives, pseudohermaphroditism, USG

## Abstract

Persistent Mullerian duct syndrome (PMDS) is a rare form of internal male pseudohermaphroditism in which Mullerian duct derivatives are seen in a male patient. This syndrome is characterized by the persistence of Mullerian duct derivatives (i.e. uterus, cervix, fallopian tubes and upper two thirds of vagina) in a phenotypically and karyotypically male patient. In this article we present the USG and MRI features of a case of PMDS with bilateral cryptorchidism and left sided inguinal hernia, containing the uterus and fallopian tubes.

## Introduction

Persistent Mullerian duct syndrome (PMDS) is a rare form of internal male pseudohermaphroditism in which Mullerian duct derivatives are seen in a male patient.[1] The syndrome is caused either by an insufficient amount of Mullerian inhibiting factor (MIF) or due to insensitivity of the target organ to MIF.[2] In this article, we present the USG and MRI features of a rare case of PMDS.

## Case Report

A 38-year-old man was found to have a uterus, fallopian tubes, and a gonad in the left hernial sac during herniorrhaphy. The patient developed a left scrotal sac swelling postoperatively.

On physical examination, the right scrotal sac was empty. The left scrotal sac appeared boggy. The patient was phenotypically male, with male pattern of external genitalia and secondary sexual characteristics. The past history included primary infertility.

**Figure 1 (a,b):**
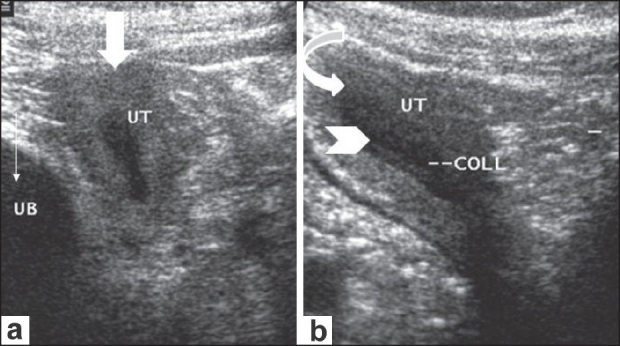
USG images at the level of the left inguinal region demonstrate the uterus (arrow in a and curved arrow in b) extending toward the left inguinal region, alongside the urinary bladder (UB in a). The endometrial cavity is distended with anechoic fluid (arrow head in b)

**Figure 2:**
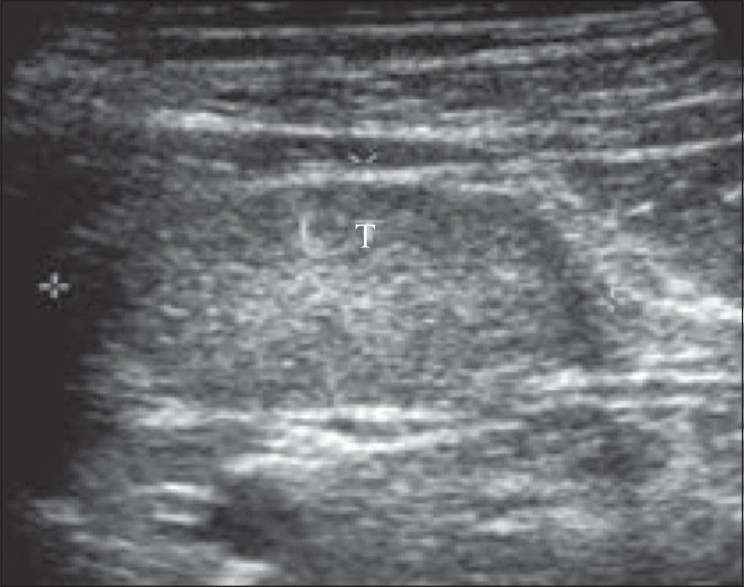
USG shows testis (T) in the left inguinal region with uniform low to intermediate internal echoes

USG showed a well-formed uterus situated alongside the bladder and extending into the inguinal canal [Figure 1a]. The endometrial cavity was distended with a small collection [Figure 1b]. An oval structure with uniform internal echoes, conforming to a testis, was noted at the level of the left deep inguinal ring [Figure 2]. The prostate and seminal vesicles were well visualized and appeared normal. The left scrotal sac showed a septate collection, consistent with a hematocele. No testis was identified within the left scrotal sac. The right scrotal sac was empty. The right testis could not be identified in an ectopic location.

**Figure 3 (a,b):**
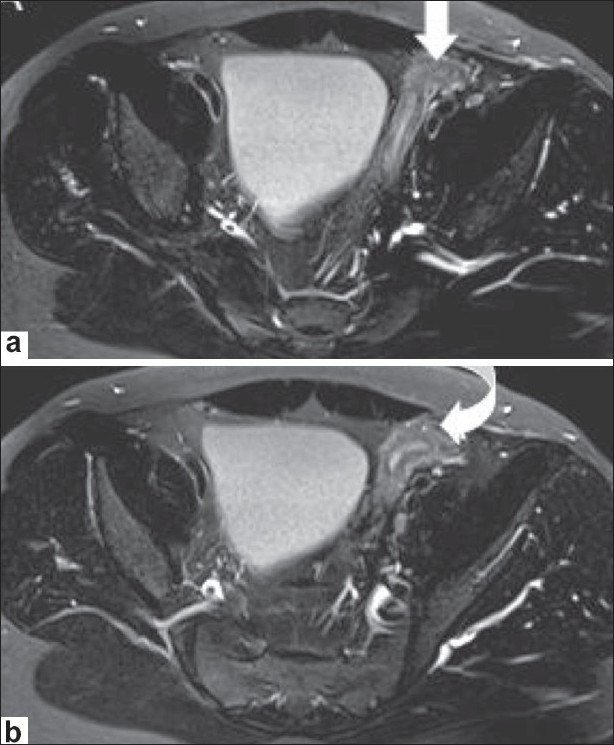
Axial T2W images at the level of the urinary bladder demonstrate the uterus extending towards the left inguinal region alongside the urinary bladder (arrow in a, curved arrow in b). The uterus shows hyperintense fluid signals within the endometrial cavity surrounded by a hypointense junctional zone

**Figure 4 (a,b):**
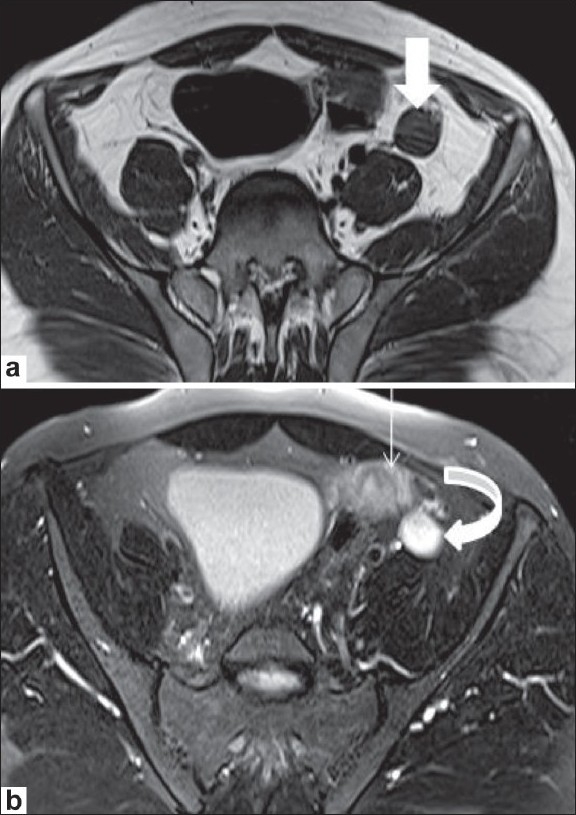
Axial T1W (a) and T2W (b) MRI images at the level of the sacroiliac joints demonstrate an oval structure with morphology and signal intensity conforming to a testis in the left inguinal region; this is isointense to muscle on the T1W (arrow in a) and hyperintense on the T2W (curved arrow in b) images. The fallopian tube (thin arrow in b) is seen along with the testis

**Figure 5 (a,b):**
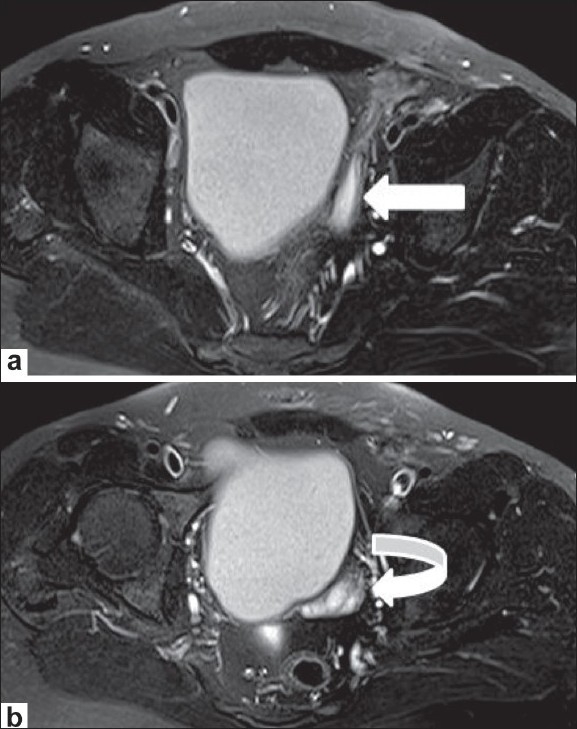
Axial T2W MRI images (a and b) at the level of the acetabulum show a vagina-like structure (arrow in a) extending toward the left seminal vesicle (curved arrow in b) in the next axial section

MRI confirmed the USG findings. It showed a well-formed uterus and fallopian tubes alongside the urinary bladder and extending into the left inguinal region [Figure 3]. A small collection was noted in the uterine cavity [Figure 3b]. An oval structure measuring 3.7 × 1.9 cm, with morphology and signal intensity consistent with a testis was detected at the level of the deep inguinal ring [Figure 4]. A vagina-like structure was seen extending from the uterus toward the left seminal vesicle [Figure 5]. No structure conforming to the testis was seen in the scrotal sac or in any ectopic location. The right gonadal vessels were seen ending blindly in the right iliac fossa, which confirmed the diagnosis of a vanishing right testis. The seminal vesicles and prostate appeared normal. No structure with ovarian morphology was seen. The collection in the left scrotal sac showed features and signal intensities of a hematocele.

Exploratory laparotomy/excision was planned for the patient subsequently. However the patient refused a second surgery.

## Discussion

PMD S is a rare form of internal male pseudohermaphroditism caused by a deficiency of MIF.[2,3] Derivatives of the Mullerian duct (i.e., fallopian tubes, uterus, and the upper part of the vagina) are present in a normal genotypically and phenotypically male. PMDS patients have normal development of external genitalia and secondary sexual characteristics.

In a human fetus the Mullerian and Wolffian ducts are both present at 7 weeks of gestation. In a male fetus, the testis differentiates by the end of the 7^th^ gestational week. Normal sex differentiation is controlled by testosterone, dihydrotestosterone, and MIF. Sertoli cells secrete MIF, which leads to regression of the Mullerian ducts. Testosterone has a direct effect on the Wolffian ducts, and promotes their differentiation into the epididymis, vas deferens, and seminal vesicles.[3] Dihydrotestosterone induces male differentiation of external genitalia. PMDS patients have both Wolffian and Mullerian duct structures due to a deficiency of MIF.

Two anatomic variants of PMDS have been described, the male type and the female type. The most common variant is the male form, encountered in 80-90% of cases and characterized by unilateral cryptorchidism with a contralateral inguinal hernia. The male form of PMDS can be of two types. The first type is hernia uteri inguinalis, which is usually characterized by a descended testis and herniation of the ipsilateral corner of the uterus and the ipsilateral fallopian tube into the inguinal canal.[3,4] The second type is crossed testicular ectopia, which is characterized by herniation of both testes and the entire uterus and both fallopian tubes.[3,4]

The second anatomic variant of PMDS, the female form, is seen in only 10-20% of cases and is characterized by bilateral cryptorchidism, with the testes fixed within the round ligaments in an ‘ovarian position’ with respect to the uterus.[3,5] The gonads are fixed within the pelvis.[4] The mobility of Mullerian structures is an important factor that determines the clinical presentation.[3,4] If the uterus and fallopian tubes are mobile, they may descend into the inguinal canal during testicular descent. On the other hand, if the Mullerian structures are relatively immobile, testicular descent may be impeded.[3,4]

In our case there was bilateral cryptorchidism, with a vanishing right testis. However the Mullerian structures were mobile and were found as part of the contents of the hernial sac. The left test is showed aborted descent and was located at the deep inguinal ring as part of the hernial sac, alongside the uterus and fallopian tube. Though our case is ambiguous and its categorization is debatable, in view of the mobile Mullerian structures, it probably falls into the first category or the male type. However, the presence of bilateral cryptorchidism could favor its categorization into the female form of PMDS.

The risk of malignancy in an ectopic testis in a case of PMDS is similar to that in a cryptorchid testis in a healthy male.[6] Germ cell tumors have been reported in the testis, whereas tumors of the Mullerian duct derivatives are very rare.[7] Since the patients are phenotypically male, the diagnosis is usually not suspected until surgery for cryptorchidism or hernia repair.

To conclude, in a case of bilateral cryptorchidism associated with hernia, as in our case, the possibility of PMDS should be kept in mind.
